# Expanding and Remixing the Metadata Landscape

**DOI:** 10.1016/j.trecan.2020.10.011

**Published:** 2020-11-20

**Authors:** Ariel A. Hippen, Casey S. Greene

**Affiliations:** 1 Department of Systems Pharmacology and Translational Therapeutics, Perelman School of Medicine, University of Pennsylvania, Philadelphia, PA, USA; 2 Childhood Cancer Data Lab, Alex’s Lemonade Stand Foundation, Philadelphia, PA, USA

## Abstract

Genomic data sharing accelerates research. Data are most valuable when they are accompanied by detailed metadata. To date, metadata are often human-annotated descriptions of samples and their handling. We discuss how machine learning-derived elements complement such descriptions to enhance the research ecosystem around genomic data.

With the past decade’s falling cost of sequencing and exponential growth of data, quantitative biology is flourishing. This growth has occurred alongside a culture of public sharing of -omic data. Integrating shared data allows researchers to attain sample sizes and statistical power that would otherwise be impossible to achieve. In turn, these lead to more sophisticated inferences that advance biological understanding. However, the value of publicly available data is most fully realized when the data are accompanied by the thoughtful and principled sharing of metadata. (We discuss the concept of metadata in more detail in Box 1.) Consider the significance of transcriptomic subtypes in high-grade serous ovarian cancer (HGSOC) as an example. While the histotypes of epithelial ovarian cancer are well defined, the extensive variation within the single histotype of HGSOC, marked by differences in survival duration, remains largely unexplained. In the past decade, numerous studies have grouped HGSOC cases into three to five subtypes, based on their transcriptomic profiles. Several subtype-specific gene signatures were associated with certain cell types, implicating cell composition as a factor relevant to HGSOC survival [1]. While there are several large transcriptomic studies of HGSOC, considering them in concert is difficult because tumor composition varies widely across existing studies [2].

## Experimental Metadata Provides Context for Data

The widespread adoption of the minimum information about a microarray experiment (MIAME) standards by journals and funders has shaped decades of metadata collection [3]. These standards were explicitly designed to dictate what information should be included about microarray data but do not specify a format for that information. MIAME compliance is impossible to effectively enforce across hundreds of journals, platforms, and repositories. This has substantially limited the *de facto* accessibility of metadata and reproducibility of experiments [4]. Subsequent efforts like minimum information about a biomedical or biological investigation (MIBBI) have expanded to encompass any potential -omics study and provide more detailed guidelines for formatting and handling metadata [5]. However, the myriad of sample-specific factors that may be relevant for the primary analysis of any given experiment has hindered metadata standardization. Considering unplanned secondary or integrative analyses further complicates standardization requirements. A recent assessment found that even basic features such as organism type and sex are not well standardized in major databases and are hard to parse with basic queries [6]. As -omic data become more central to the biomedical research ecosystem, journals and funders need to consider the impact of high-quality sharing when making editorial and funding decisions [7].

Standardizing metadata for existing experiments is a monumental task. Ontologies can be helpful. Some, including the Experiment Factor Ontology, provide protocol-related information, while others capture terms related to healthy and malignant tissues. The Ontology Lookup Service provides an interface to find terms and relevant ontologies. Manual metadata curation in small, localized contexts, such as the curatedOvarianData R package, provides great value to researchers working entirely within HGSOC [8]. But manually curating entire databases of sequencing results is infeasible. We expect computational approaches will begin to supplement user-supplied metadata and indeed become the predominant means of sample annotation for secondary analyses. Automated text-mining workflows, such as those in metaSRA, can standardize written sample descriptions [9]. However, elements never described within the text remain unrecoverable. We anticipate that advances in metadata inference will complement text mining to greatly expand the available computationally derived metadata.

## Omics-Derived Metadata Complement Standard Annotations

Using computationally derived metadata is not a new approach: in human genetic studies, controlling for population genetic differences is essential. Many studies using biobanked samples have limited access to the type of detailed ancestry information that would be required: instead, investigators usually have self-reporting into one of a few discrete ethnicity categories. Decades of research have clearly shown that even sparse genomic profiles capture population stratification with more detail and nuance than most studies can directly gather [10]. Many genetic studies now use ancestry principal components to control for and assess population differences [11]. For other types of -omic profiles, examining ancestry with such detail has been impossible. However, as publicly available -omic profiles shift to next-generation sequencing, it becomes increasingly possible to call variants for other types of profiling as well. There remain challenges to address: different types of -omic profiling methods have different error rates in different regions and contexts and principal component analysis is wholly dependent on the dataset used, so study-specific principal components would not be useful in a meta-analysis. However, projecting samples onto principal component values from a standard reference such as 1000 Genomes could be widely adopted to provide inferred ancestry information to studies that lack it, including RNA-seq experiments, and enable broad meta-analysis.

We are witnessing the emergence of methods to infer metadata beyond ancestry from only primary sequence or other -omics data. These methods can compensate for missing metadata and provide valuable new annotations to support secondary analyses. For instance, the creators of the recount2 database used well-annotated public data to develop a phenotype predictor and annotate essential metadata such as sex and tissue type with high accuracy [12]. Applying this predictor to public sharing databases is much more scalable than manual or semi-manual curation, so it is reasonable to imagine that these predictions could become acceptable as a proxy or even a replacement for user-curated metadata. These predictions could be supplied with probability or accuracy estimates. Derived -omics metadata can also provide insight above and beyond recovering missing values from typical metadata. For example, DNA methylation has been described as a means to measure aging, a supplement to chronological age data known as ‘biological age’, which has been associated with cancer risk [13]. Models trained on methylation or multi-omic data may benefit from including biological age as a covariate and could support or refute associations with other phenotypes.

In transcriptomics, heterogeneity in cell composition has long been a confounding factor. Deconvolution allows for computational estimation of cell type proportions in bulk RNA-seq data [14]. Controlling for differences in cell type prevalence across samples in a study or across studies enables researchers to find more subtle differences in expression signatures and better understand underlying biology. Many questions centered on the tumor microenvironment are addressable with this method, including questions related to HGSOC transcriptomic subtypes. A resource providing estimates of cell type proportions in each tumor, derived from secondary analysis of curatedOvarianData using multiple deconvolution methods, could support tertiary analyses examining cell type associations with exposures, outcomes, and other annotated features by a broader community of interested investigators. Controlling for stromal cell composition could uncover differential expression in important cancer pathways, suggesting targets for subtype-specific therapies.

## Storage and Sharing Solutions Are Required for Omics-Derived Metadata

It is clear that data without context are less valuable than those with it. As we look ahead, we expect both the need for computationally derived metadata and our ability to generate such metadata will increase. With MIAME and MIBBI, the field encouraged metadata sharing but did not standardize structure, which has limited the extent to which metadata support further analysis. Numerous methods are being developed that offer improved and novel ways to contextualize primary data. Some are akin to those examining ancestry, sex, and other factors that could be well standardized into ontologies *a priori*. Others are unsupervised and capture some factors that may be well understood and others that many only be understood after subsequent research. For example, MultiPLIER derives latent variables from large gene expression compendia, but the interpretation of these latent variables may only occur after the fact [15]. This is an active area of research and we should anticipate new methods for metadata inference. It will be important to consider how metadata with confidence levels, such as an annotation of an ontology term and a probability, can be appropriately handled.

**Figure 1.**
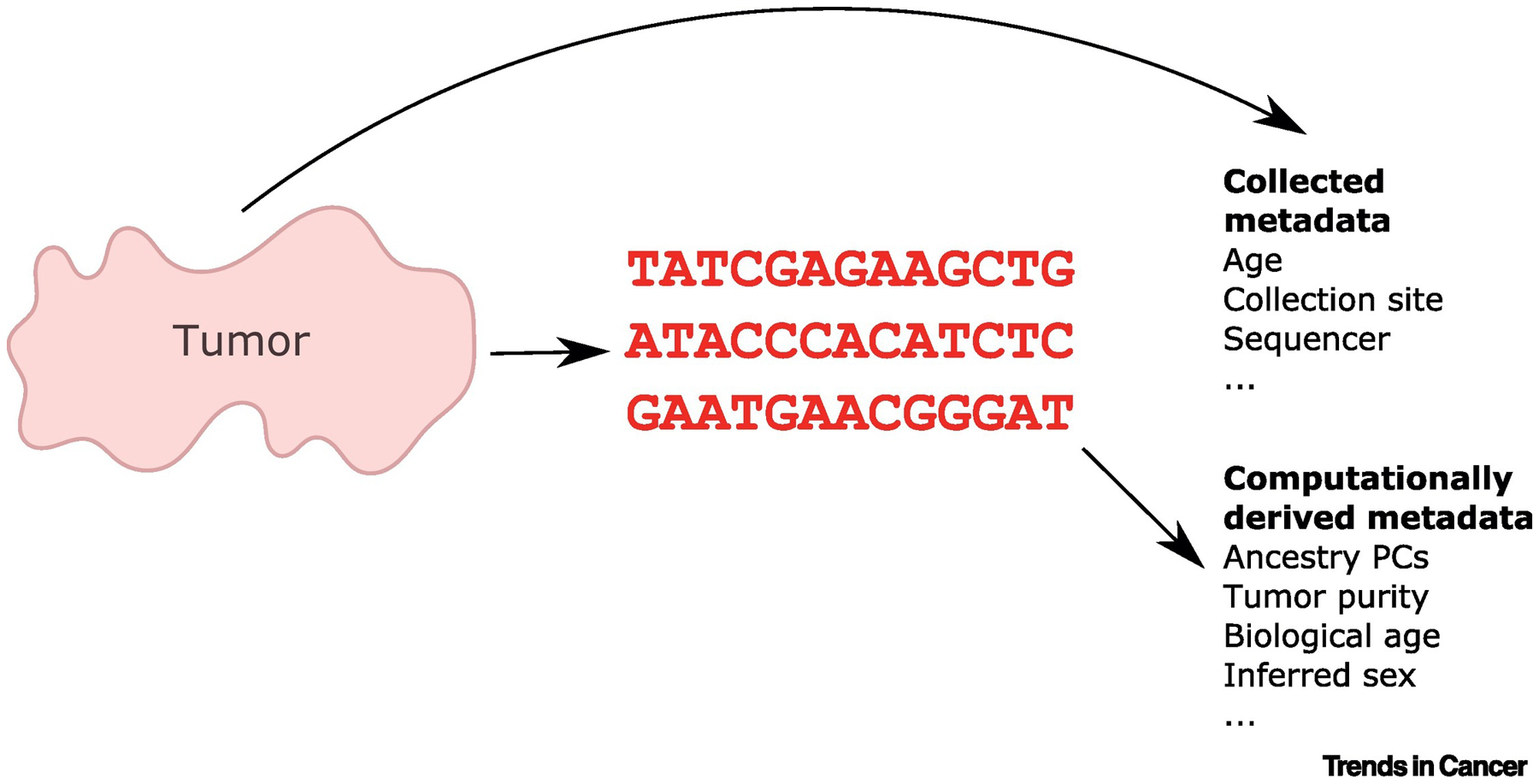
A Schematic of Sample Characteristics and How They Relate to Metadata for a Hypothetical Piece of Tumor Tissue. Metadata can include elements curated from records that describe a patient or those related to the process of sample collection and quantification. They can also include elements estimated from sequencing data, which may include elements like biological age that are not directly observable or extractable from existing records. Abbreviation: PC, principal components.

Now is the time to carefully consider how we can design resources and incentives to increase the extent to which computationally derived metadata (Figure 1) will support tertiary analyses. Transcriptomic and other -omic data provide a molecular snapshot of the system under study. In some cases, it can be possible to infer context directly from such data. In other cases, it is only possible to identify a source of variability but not to ascribe it to underlying properties of the sample. Detailed annotations of what biological conditions the data represent and how they are measured foster better meta-analyses. It is particularly important that metadata, regardless of how they are derived, be managed with an authoritative single point of truth just like data [7]. One of the principal needs is a single point of truth metadata repository designed to store and share metadata created from one or more studies from multiple sources, which may or may not include the investigators who initially deposited the data. We envision a database where computationally derived metadata could be shared in a standardized way and secured in cases where risks require it. Each metadata record could include bidirectional links with the primary data sources in (for instance) ArrayExpress or SRA, structured information about any transformations done, and results from one or more metadata-producing algorithms with annotations supplied to well-structured ontologies where possible. Over time, these would enable tertiary analyses where users could aggregate results from computed metadata to better understand underlying disease processes.

## Box 1. What Are Metadata?

The term metadata refers to data that describe data. These can describe a sample, such as the biomaterial the data were derived from: cell line or tissue, disease case or control, or any experimental interventions made, like drug dose. They can also describe how a sample was handled, including processes for sample purification, profiling, and quantification: for instance, any dissociation protocols, sequencing machine, and normalization steps. Metadata for sample handing is key for performing batch effect correction and other technical steps to correct for artifacts. Traditionally, metadata would be annotated at the time of sample processing. It is now possible to produce computationally derived metadata annotations by inferring metadata elements. These elements, which may be the primary outputs of one analysis, could then be used as covariates in future analyses. When annotating metadata, it can be helpful to identify standardized terms, such as those available from an ontology lookup service. It is ideal to store metadata in a repository that provides a single point of truth. The idea of a single point of truth comes from software development, where it is key to have one trusted location with which others can be compared.
